# Supplementary material for: Understanding Inequalities in Ride-Hailing Services Through Simulations
Source: Sci Rep. 2020 Apr 16;10:6500. doi: 10.1038/s41598-020-63171-9 (PMC7162900; doi:10.1038/s41598-020-63171-9)
Supplement: Supplementary file 1 — Supplementary Information. [file 41598_2020_63171_MOESM1_ESM.pdf]

Supplementary Information for the paper Understanding Inequalities in Ride-Hailing Services Through Simulations

Eszter Bokányi[a,b] and Anikó Hannák* [c,d,e]

[a] *Eötvös Loránd University, Budapest, Hungary*
[b] *Agglomeration and Social Networks Lendület Research Group, Centre for Economic and Regional Studies of the Hungarian Academy of Sciences*
[c] *Complexity Science Hub, Vienna, Austria*
[d] *Vienna University of Economics and Business, Austria*
[e] *Centre for Economic and Regional Studies of the Hungarian Academy of Sciences*

Robustness

We include results demonstrating robustness checks in the following four areas:

1. Pick-up and drop-off location distributions: We show that the effect of the initial function we use to create the pick-up and drop-of location distributions is negligible. We compare the 2D Gaussian function used in the paper with a uniform, sigmoid, and an exponential setup.

2. Differences between different runs of the same simulation: we find that the size of the variance between multiple identical simulations is negligible.

3. Different city size: We compare the 20*20 grid size used in the paper with a 100*100 grid.

4. Simulation results over time: we show how income distributions change over time as we run our simulation for 10 weeks.

*To whom correspondence should be addressed. E-mail: aniko.hannak@wu.ac.at

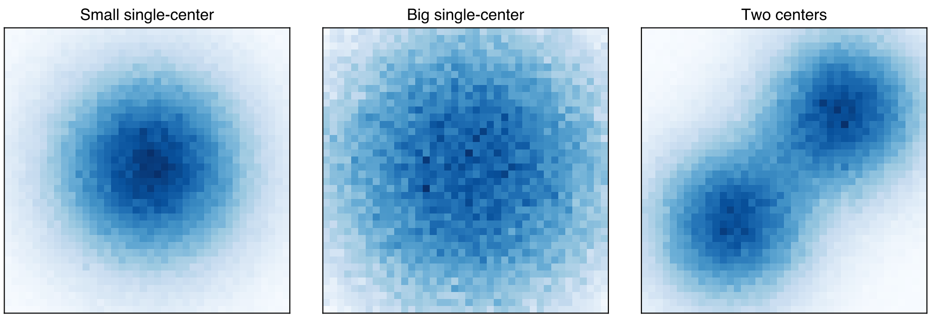

(a) Symmetrical cases, Gaussians

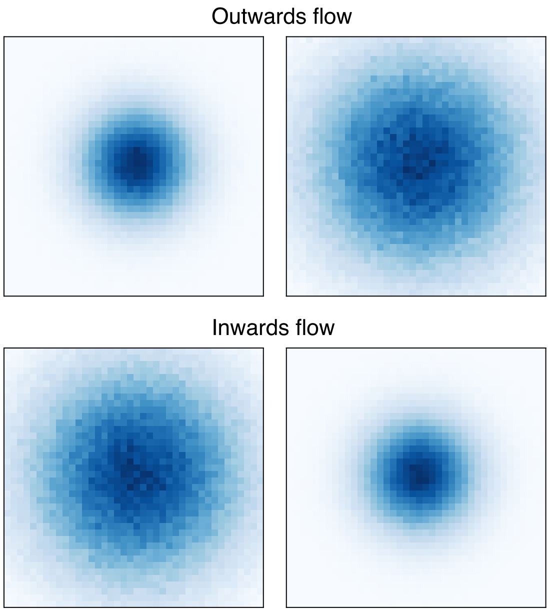

(b) Traffic flows, Gaussians

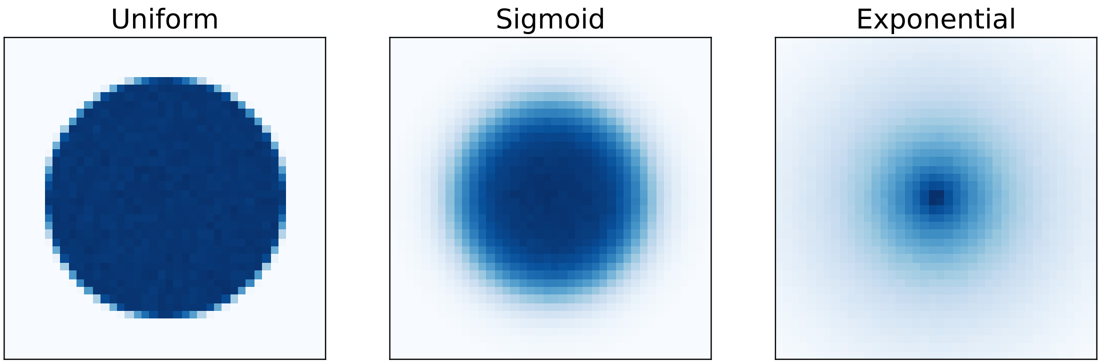

(c) Other distributions

Figure 1: Distribution of pick-up and drop-off locations used in our simulations. (a) Symmetrical Gaussian cases, where pick-up and drop-off distributions are identical. (b) Pick-up Gaussian on the left and drop-off Gaussian on the right, where the two distributions do not overlap. (c) Alternative distributions tested for trip length and income distributions: uniform, sigmoid, and exponential, respectively.

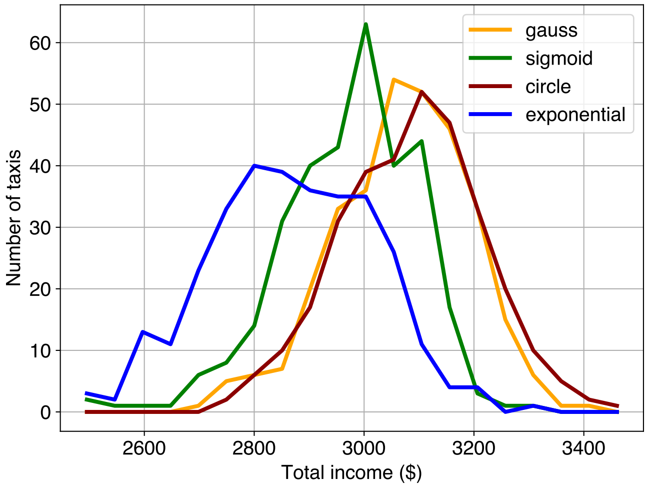

Figure 2: Income distributions for $d = 2.1$ [$1/\text{km}^2$] and $R = 0.5$ for the different single-center geometries from Figure 1a left, and Figure 1c. Pricing scheme differs from that of the main article, therefore, we can only address the distributions qualitatively. The only significantly different shape is that of the exponential distribution, that has 0.29 Gini index, whereas the other three distributions all possess a Gini of 0.23.

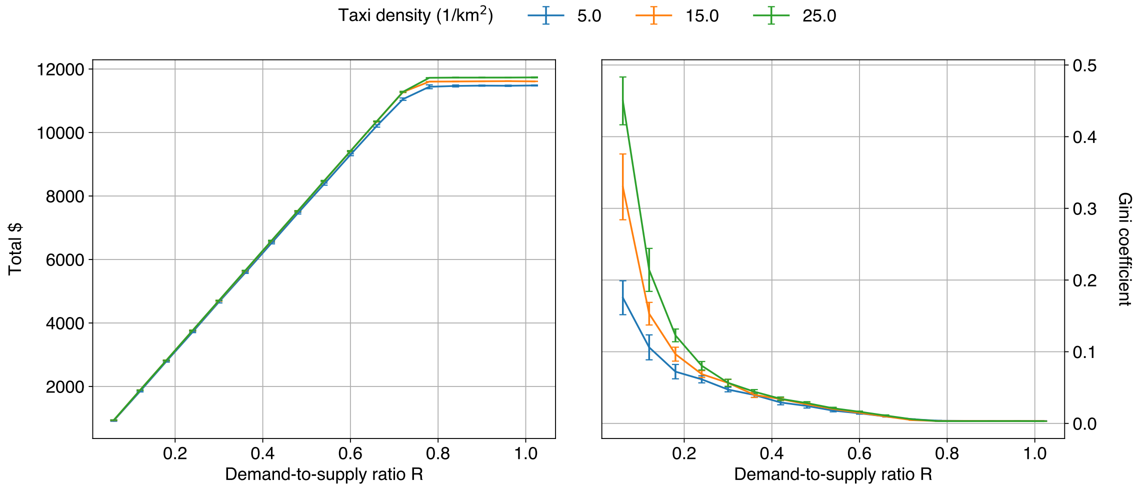

Figure 3: Figure 1B-C of the main article with error bars showing the variation between simulation runs in the average driver incomes and the Gini indices. The simulations yield almost the same results, the variation in average earnings is almost negligible. Though for small R, the variation in Gini is higher than for high R, the differences and trends are clear even when the errors are included.

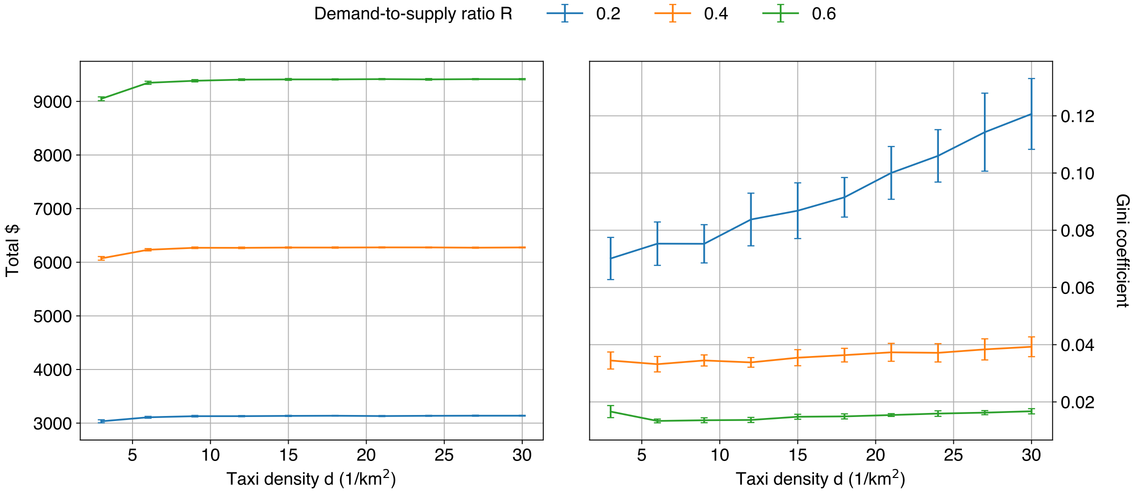

Figure 4: Figure 1D-E of the main article with error bars showing the variation between simulation runs in the average driver incomes and the Gini indices. The simulations yield almost the same results, the variation in average earnings is almost negligible.

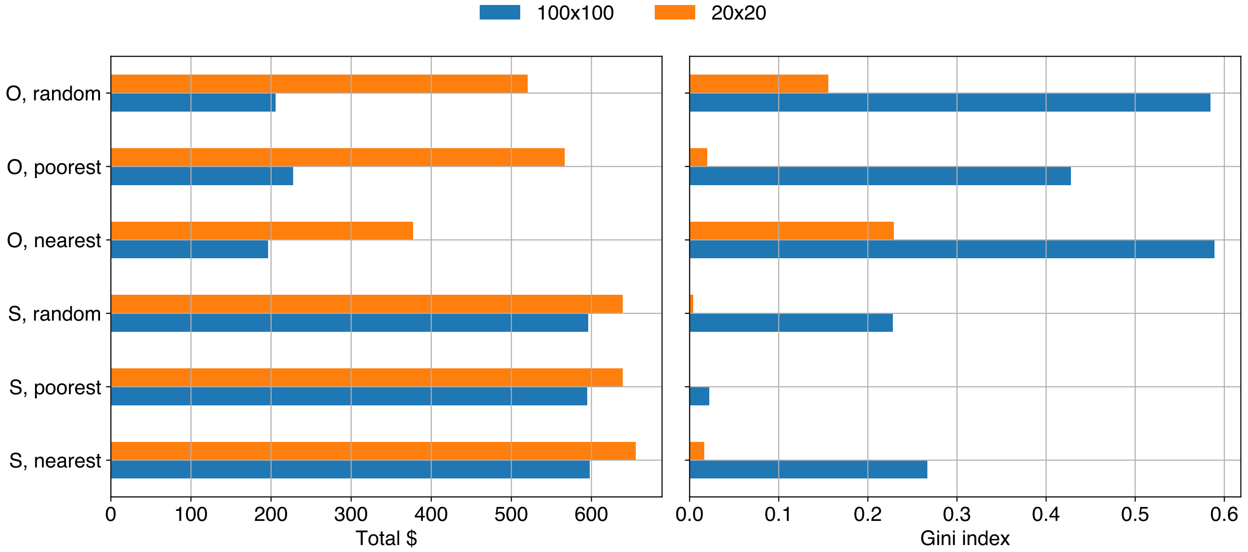

Figure 5: Comparison of average incomes and Gini indices in two simulated cities of different sizes at $d = 2.1$ $[1/\text{km}^2]$ and $R = 0.5$ for two city layouts and all three algorithms. Orange corresponds to the main article's simulated 20×20 grid, blue to a 100×100 grid, that corresponds to 100 km^2 in SI units. 'S' denotes the Small single-center city layout, 'O' denotes the Outwards flow city layout, algorithms are indicated next to the layouts. Average incomes decrease because in a bigger city, taxis tend to have more empty kilometers. Though Gini indices increase significantly, the qualitative differences between algorithms and geometries are kept after rescaling the simulation to a bigger city. It means that the Outwards flow city layout creates a more inequal distribution than the Single center layout, and that the ranking of the algorithms according to fairness is *poorest*, *random*, and *nearest* in increasing order.

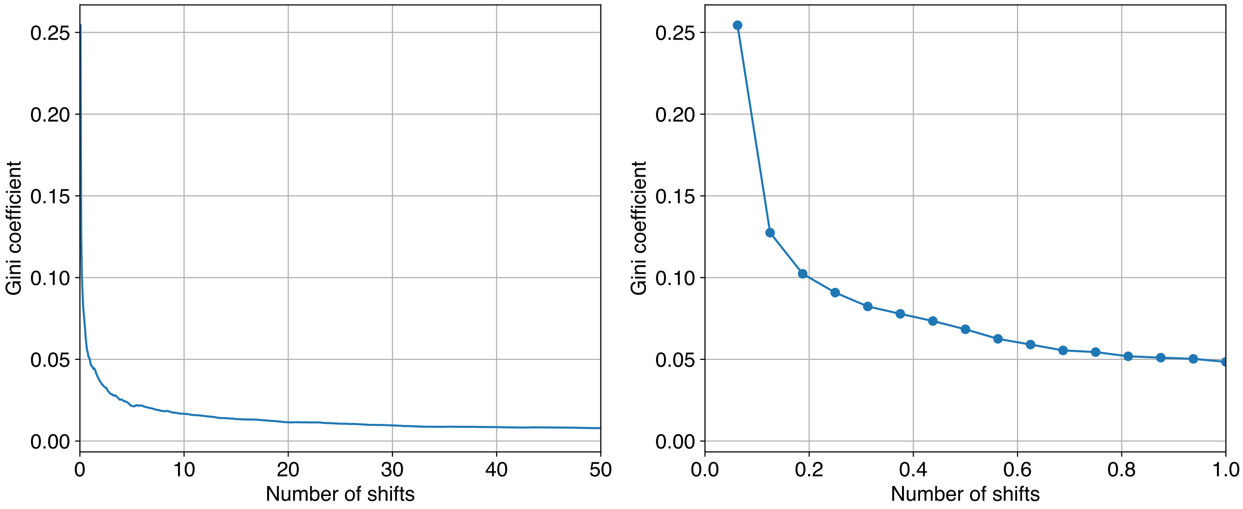

(a) Time series of Gini

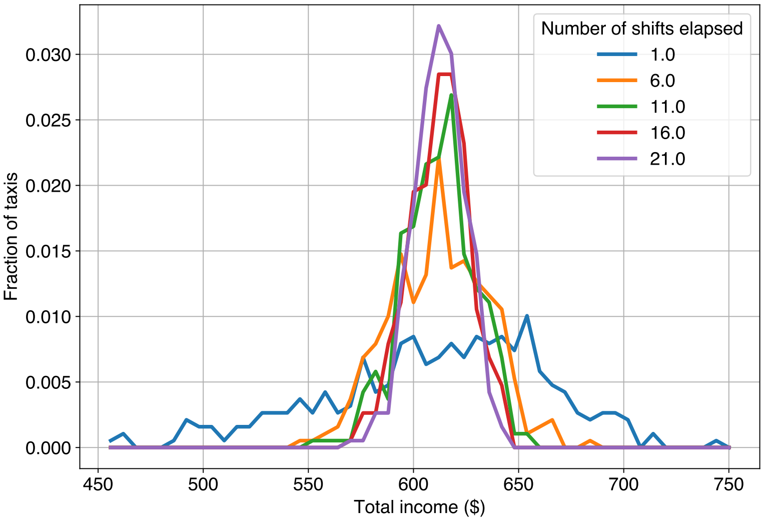

(b) Income distribution evolution

Figure 6: Changes in Gini index (a) and income distribution (b) with simulation time for the nearest matching algorithm, with $d = 2.1$ $[1/\text{km}^2]$ and $R = 0.5$, small single-center city layout. (a) Gini index decreases with simulation time, as income distributions become sharper. The initial sharp decrease, it is followed by a slower decrease, that indicates that the timescale on which inequalities would ultimately disappear is large. (b) The shape of the income distribution after a given number of 8-hour shifts. Even after one month (21 shifts), there might remain 5-10% differences in driver earnings.
